# Social remittances during COVID-19: on the “new normality” negotiated by transnational families

**DOI:** 10.1186/s40878-021-00263-z

**Published:** 2021-11-09

**Authors:** Nare Galstyan, Mihran Galstyan

**Affiliations:** Ethnosociology Department, Institute of Archaeology and Ethnography, National Academy of Sciences of the Republic of Armenia, Yerevan, Charents str. 15, 0025 Republic of Armenia

**Keywords:** Social remittances, Transnational families, COVID-19 (coronavirus) pandemic, Pandemic transnationalism

## Abstract

Social remittances- the transfer of ideas, practices, and codes of behaviors- are a well-documented subject in migrant transnationalism literature and transnational family studies. However, the COVID-19 (coronavirus) outbreak has generated unprecedented regulations around the world which require widening the conceptual basis of social remittances in a state of emergency. As the pandemic progresses, most countries require civilians to follow a number of norms deemed as the “new normality,” while other countries continue to operate under their “old normality,” with minor or no restrictions. As national pandemic policy responses vary across countries, transnational families live under different regimes of “normality.” In these settings, the study of transnational families offers a valuable opportunity to examine a special type of social remittances practiced during global crises, and analyze whether the exchange of rules, practices, and ideas across national borders has any impact on re-shaping and renegotiating pandemic-coping “new normality” practices for both migrants and their families. The paper is based on 13 in-depth interviews conducted with representatives from Armenian transnational families with migrant members in Russia, the Czech Republic, or Belarus. These countries provide a backdrop for an examination of social remittances among transnational families that we term “pandemic transnationalism.” The study shows that the circulation of safety rules and “good practices” actively shapes the everyday behavior of migrants and their families, their level of perceived danger towards the coronavirus, and their practical knowledge of safety measures. The latter are often harnessed in informal collective settings despite being in conflict with the obligations and regulations of their home society.

## Introduction

In this article, we provide an original contribution to the academic debate on social remittances as an inseparable part of transnational families’ lives during pandemics. Since the outbreak of the COVID-19 (coronavirus) pandemic, academic literature has been increasingly enriched with studies that reveal the links between the spread of the COVID-19 disease and migration. A significant share of the literature has focused on the impact of the pandemic on financial remittances sent by migrants (Diao & Wang, 2020; Furceri et al., 2020; Murakami et al., 2020). Just as when the concept of “social remittances” was introduced by Levitt (1998) a little over two decades ago, it is important to underline today that in addition to money, migration leads to the circulation of ideas, practices, skills, identities, and social capital between sending and receiving communities.

In addition to the general guidelines provided by the World Health Organization, countries developed their own strategies and preventive measures to cope with COVID-19. The term “new normality” underlines the long-term effects of the pandemic on nearly every aspect of human lives. Transnational families are especially vulnerable under these conditions as migrants and their families left behind in home-countries find themselves embedded in systems with different approaches to coping with COVID-19. This situation provides opportunities to question the role and characteristics of social remittances in times of global crisis, as well as the extent to which the exchange of rules, practices, and ideas across national borders re-shapes and re-negotiates pandemic-coping “new normality” practices for both migrants and their families.

The study is based on 13 in-depth interviews with Armenian transnational families with migrant members in Russia, the Czech Republic, or Belarus. Our current study contributes to existing migration literature by focusing on a special type of social remittances practiced during global crises that we term “pandemic transnationalism”: the circulation of ideas and practices in times of pandemic which encompasses an exchange of informal practices that affects not only the lives of migrants and their families in their home country, but their close circle of friends and neighbors as well. The study highlights the potential challenges of negotiating social and cultural norms within “new normality” guidelines provided by both the World Health Organization and sending and receiving states. The direction of social remittances is another important aspect of “pandemic transnationalism” that the paper unpacks, as reverse social remittances informally regulate the lives of people across borders and impact the pandemic coping strategies chosen by transnational families.

To develop these ideas, we first review the literature on social remittances with references to the literature on transnational families. The next section provides the methodological background of the research. Following a discussion of the migration context in Armenia, we present a brief introduction to the COVID-coping strategies of the interviewees’ family members’ countries of residence. Next, we discuss the circulation of pandemic-coping strategies, and the interactions between formal guidelines and informal practices negotiated in transnational families. In the last section, we discuss the social and cultural norms that affect the ability of transnational families to live under the “new normality.”

### Conceptual basis: is there a knowledge gap on social remittances in times of global crisis?

It has been long-established in migration literature that migrants do not abandon their links with their country of origin when moving from one country to the other, but sustain social relations, interactions, and formal and informal links that transcend national boundaries (Ambrosini, 2018; Blanc et al., 1995; Faist, 2013; Özveren & Faist, 2004; Kivisto, 2003; Waldinger & Fitzgerald, 2004). There is a wide range of migrant transnational relationships, including dual citizenship, family connections, hometown associations, and community connections across multiple nation-states (Faist, 2000). Through transnational ties, migrants and non-migrants circulate both material and immaterial goods that can directly or indirectly impact both sending and hosting societies (Boccagni & Decimo, 2013; Isaakyan & Triandafyllidou, 2017; Levitt, 1998, Levitt, 2001; Sasse, 2013). The phenomenon of circulating immaterial goods has largely been referred to as “social remittance.” Levitt (1998) conceptualized a well-known definition of social remittances that will be used in the scope of this study: ideas, behaviors, identities, and social capital that flow from receiving to sending country communities. Levitt and Lamba-Nieves (2013) find that these practices circulate interpersonally between individuals who learn of, adapt, diffuse, and sustain ideas and practices through family and kin networks. Levitt and Lamba-Nieves (2013) suggest that the dynamics of social remittances are marked by a complex interplay between the migrant, the host society, and the country of origin.

“Social remittance” has often been used as an umbrella term to discuss various aspects of migrants’ non-monetary transfers. Social remittances have been documented in the realm of political remittances, social aspects of economic remittances, and cultural remittances. Political remittances encompass the potential of migrants to cause political changes through transferring political ideas and practices, as well as narratives of belonging in places of destination and origin (Adamson, 2020; Brinkerhoff & Taddesse, 2008; Krawatzek & Müller-Funk, 2020). Another stream of literature has focused on social aspects of economic remittances as mediators of relationships between individuals and groups across borders (Biggart & Castanias, 2001; Paerregaard, 2015; Singh et al., 2012; Sørensen et al., 2003; Thai, 2006). Considerable literature has been produced on cultural remittances, the diffusion of cultural values and norms from host-country to home communities. New norms taken from the host society can be transferred back home through family, social, and community networks (Bell & Bivand Erdal, 2015; Goździak et al., 2020; Hanifi, 2006; Levitt, 1998). Even though the concept of social remittances covers an extremely diverse set of phenomena, we suggest addressing it as a circulation of ideas and practices that affect everyday lives, values, attitudes, and practices both in home and host countries by embedding it in the study of transnational families.

The basic unit through which ties are maintained and ideas are circulated within the home country is the family: siblings, parents and children, and grandparents and grandchildren. Migrants and non-migrants continue to belong to a family unit even though they may not see each other or be physically separated for extended periods of time. As Baldassar et al., (2007, p. 13) noted, family members retain their sense of collectivity and kinship through “transnational families” in spite of being spread across multiple nations. Several studies have underlined that the maintenance of significant ties between migrant and non-migrant members of the same household entails renegotiation of family dynamics (Baldassar & Merla, 2014; Dreby & Adkins, 2010; Levitt & Schiller, 2004). Furthermore, in everyday life important family decisions are often made through long-distance communications by constant redistribution of roles and duties (Yeoh et al., 2005). In the process of exchanging values and norms many families go through transformation, such as a change of gender roles, delegation of household tasks, organization of family tasks, and more (Parreñas, 2001; Suksomboon, 2008). An inseparable part of the transnational family relationship is care-giving or “kin-keeping” practices and responsibilities (Ackers & Stalford, 2004). The latter may take the form of providing small favors, money, advice, organizing family holidays, return visits, phone calls, and more. Based on Caribbean and Italian family life, Zontini and Reynolds (2007) suggest that family members devote considerable time and energy to this, despite the geographical distance. Exchange of caregiving is a key feature of the “doing family” practice (Morgan, 1996). Solari (2019) uses the term “transnational moral economies” to define the ways in which parents and children within transnational families create dynamic meaning-making systems that link economic and social practices. The exchange of social remittances helps to reproduce and maintain family relationships and hold family members together as a part of a common unit. Other studies on social remittances have predominantly discussed it within the migration–development nexus with a focus on sending social remittances from developed to less developed countries, and on migrants as brokers of changes introduced in their households, communities, and homelands (Garapich, 2016; Grabowska & Garapich, 2016; Vianello, 2013).

At the moment, when it seems that much has been said in the literature on social remittances, the ongoing COVID-19 outbreak urges scholars to readdress the characteristics of social remittances in times of crises. Studies on remittances in times of crises have been mainly limited to political remittances in times of conflict, while a large body of literature has focused on financial remittances following global crises (Chami et al., 2010; Murakami et al., 2020; Ratha & Sirkeci, 2010; Sirkeci, 2020). Since the outbreak of the COVID-19 pandemic, a few studies have uncovered health, economic, social, or political changes for migrants amid the ongoing pandemic (Abel & Gietel-Basten, 2020; Ang & Opiniano, 2020; Bukuluki et al., 2020). Studies have underlined the everyday effects of the pandemic on the lives of people, and the pandemic has also compelled scholars to look at transnational caregiving practices within families. The role of the family in protecting its members from COVID-19 has been deemed important in developing individual and collective resilience (Walsh, 2020). Nevertheless, the ways in which pandemic-related practices travel and are negotiated among two shores of transnational families have not received sufficient scholarly attention.

Another theoretically underdeveloped issue that needs special attention is the direction, initiators, and receptivity of social remittances during crises. Studies on social remittances have mainly focused on migrants’ ability to accelerate and modernize development in their countries of origin (Inglehart & Welzel, 2005) and contribute to the development of less-developed countries (Isaakyan & Triandafyllidou, 2017; Levitt & Lamba-Nieves, 2013). Highly-skilled migrants play an important role in this process (Isaakyan & Triandafyllidou, 2017; Sturge et al., 2016), whom Levitt (1998, p. 931) refers to as “purposeful innovators” that “aggressively search for, select and absorb” new practices. Studies show that non-migrants are not simply the consumers of ideas and practices sent from abroad. Non-migrants often show low receptivity, and resistance or adaptation of the transmitted values to the local context, as social remittances are not always in line with local perspectives (Dzięglewski, 2016; Grabowska & Garapich, 2016; Levitt & Lamba-Nieves, 2013; Nevinskaitė, 2016).

The directions of social remittances during the COVID-19 pandemic are of special importance considering that unlike other forms of crises, such as conflicts or economic crises, senders are affected by the same event as the recipients, even if they reside in other parts of the country or overseas. The importance of reverse remittances as a cross-border phenomena has been largely highlighted by Mazzucato (2011). Reverse remittances are material, emotive, and symbolic resources that flow from home to migrants abroad (Ambrosini, 2018; Boccagni, 2015; Mazzucato, 2011; Yeboah et al., 2019). Reverse flows of social remittances, as well as resistance to and adaptation of social remittances sent from abroad during a pandemic are crucial aspects to consider for a complete understanding of the reciprocity of sender-receiver relations.

### Migration context of transnational families in Armenia

The Armenian-Azerbaijani conflict over Nagorno-Karabakh, the economic crisis, high unemployment, low wages, and political instability have been the main factors influencing Armenian emigration since the collapse of the Soviet Union. According to statistical data, during the past 20 years more than a million people emigrated from Armenia. From just 1988 to 1995, 800–900,000 people emigrated from Armenia, or 25% of the total population (IOM, 2008), and from 1996 to 2001 around 250,000 people migrated abroad, mainly due to family reunification policies (NSS of RA, 2002). Following socio-economic stabilization, the average number of migrants was estimated to be 20,000 people emigrating per year from 2002 to 2011 (Statistical Committee of RA, 2019). From 2012 to 2014, the average number of emigrants was approximately 72,000 people per year, and from 2015 to 2017, 40,000 people per year (NSS of RA, 2017). In 2018, 15,300 people migrated abroad, the vast majority of which (69.5%) migrated to the Russian Federation, 1.5% to other Commonwealth of Independent States (CIS) countries, 6.5% to European countries, and 3.4% to other countries (NSS of RA, 2019).

Due to migration from Armenia, families whose relations are formed “at a distance” have become widespread. The consequences of family separation are immense, including divided family relationships, negotiation of family roles at a distance, challenges of family reunification that occurs only 2–3 months per year and more (Galstyan et al., 2017). Transnational families have a distinct gendered character, as 91.3% of migrants are men, while women generally stay “back home” (Statistical Committee of RA, 2019). In a few cases, children are also divided, with one child living with his or her parents abroad, and another living with his or her grandparents in Armenia. Migration has resulted in several social changes in traditional family models, such as changes in cultural norms and new roles for women (Galstyan et al., 2017). The context of transnational families in Armenia opens a wide field to study the exchange of social remittances during a pandemic.

## Research methodology

The article is based on qualitative research conducted among Armenian families who have members living abroad during the COVID-19 pandemic. Qualitative methodology was applied to grasp subjective interpretations of feelings and meanings behind social remittances. This approach helped to problematize loyalties to official guidelines on “new normality”, and study less formal pandemic-coping strategies. Through in-depth interviews, it was possible to generate an understanding of narratives on how transnational families coped with the coronavirus, with special attention given to family narratives from February 2020 to October 2020. We asked questions about the information and practices circulated between the migrant and non-migrant members of households, perceived dangers and anxieties towards the pandemic, and how pandemic-coping practices have been negotiated and changed within the selected families.

The research included13 in-depth interviews with non-migrants residing in Armenia. The interviews were conducted with representatives of Armenian transnational families that have migrant members in Russia (5), the Czech Republic (5), or Belarus (3). Selecting families that had migrant members in different countries provided a more rounded and comprehensive understanding of the characteristics of social remittances in times of pandemic, and their alignment or conflict with official strategies of coping with the outbreak. Included among the selected families are low-skilled migrants (6) and high-skilled migrants (7). This approach was selected as the literature on social remittances places significant attention on high-skilled migrants that play the crucial role in transferring ideas and practices (Holdaway et al., 2015; Wong & Levitt, 2014), while low-skilled migrants are believed to affect “basic transnationalism,” mainly restricted to the family- and household-levels (Ambrosini, 2018). The interviews included people who had survived being infected with the coronavirus and those who had not been infected. The interviewed families (non-migrants) reside both in urban areas, such as the capital city of Yerevan, and in rural areas of the Gegharkunik region, which includes one of the higher number of migrants in Armenia (NSS of RA, 2019).

### Covid-19 response in Armenia, Russia, Belarus, and the Czech Republic

In 2020, COVID-19 moved rapidly across the world despite many countries perceiving the outbreak as a far-off threat. While most national COVID-19 protective measures have followed the World Health Organization’s standards, policies and practices of coping with the pandemic can vary. Russia, Armenia, and Belarus share a common Soviet past and similar health systems, and were expected to implement similar responsive measures (Åslund, 2020). In all three countries, the first cases of coronavirus were found in February 2020. Belarus’s government demonstrated a complete denial of the COVID-19 pandemic, as all mass gatherings, such as the Belarusian Victory Day parade, took place without any delays (Dellanna, 2020). In a now-famous speech, President Aleksander Lukashenko openly expressed his skepticism towards the pandemic by asking if anyone could demonstrate that coronavirus existed, and suggested that people drink vodka and go to the sauna to stay healthy and stave off the virus (Haltiwanger, 2020). The policy of skepticism hasn’t changed in Belarus since the start of the pandemic, and until now no special measures or lockdown have been imposed (Kramer, 2020; Khurshudyan, 2020).

Overall, pandemic policies in Russia and Armenia appear similar. Both Armenia and Russia denied the seriousness of the virus for a long time by playing down its possible health impacts. Vladimir Putin, the president of Russia, continued to hold meetings without masks, and similarly in Armenia Prime Minister Nikol Pashinyan held mass meetings for a constitutional referendum (MassisPost, 2020). However, both Russia and Armenia changed their approach and started taking the pandemic more seriously since March 2020: both countries declared a state of emergency since March 2020, mainly coordinating their actions with the standards of the World Health Organization. The fight against coronavirus was conducted through the following mechanisms: isolation, wearing masks, disinfection of hands, maintenance of physical distance, restriction of free movement, and prohibition of large events. Although the Armenian government initially implemented strict control mechanisms, the large influx of labor migrants (mostly from Russia) allowed the virus to spread (Aslanyan & Mirzoyan, 2020). As a result, Armenia was among the top ten countries in the world in terms of COVID-19 cases per capita (World Health Organization, 2020a, b). Due to gradual improvements, the state of emergency was replaced with a state of quarantine on September 11, 2020. Restrictive measures were relaxed due to the outbreak of war over Nagorno-Karabakh on September 27, 2020 and haven’t been reinstated since then (Aslanyan et al., 2021).

In the Czech Republic, stricter safety measures were introduced by the central government at the end of February 2020. The state of emergency or “blanket quarantine” as it was termed in the Czech Republic was declared on March 12, 2020. The strict restrictive measures worked quite well during the first phase of the pandemic in the Czech Republic, to the point that around 2000 people gathered at a 500-m-long dinner table on Charles Bridge in Prague for a party billed as a “symbolic farewell” to “the crisis” in June (Shotter, 2020). The slow-down in the spread of infection was attributed to the initial government response to the coronavirus. Despite this, the Czech Republic tightened lockdown measures later on, as the government battled the world’s worst surge in COVID-19 infections (Kottasová, 2021).

The study revealed that the attitudes of migrants’ families in Armenia towards the pandemic measures in Russia, Belarus, and the Czech Republic varied. According to the interviewees, Armenia, Russia, and, to some extent Belarus, have more similarities than differences in the fight against the virus. Migrants living in those countries shared with their families in Armenia a number of stories regarding how their governments long-denied the danger of the virus. The attitude towards the coronavirus was largely summed up as “something like a flu that will pass soon.” A few respondents claimed that the ineffectiveness of the fight against coronavirus was influenced by cultural practices established in Soviet Union for 70 years: a lack of discipline and responsibility, as well as distrust towards state information channels. Sasha, a 76-year-old man, shared his perception:

In the 1990s, when the Soviet state collapsed, I emigrated from Armenia with my family, and lived in Russia for about 20 years. Then I lived in Belarus for 8 years because my daughter was married there. Now my son lives in Russia, my daughter in Belarus, and my wife and I have returned to Armenia after retirement … We can overcome COVID-19 only with discipline and civil responsibility. It is still lacking in Russia, Armenia, and Belarus, because my generation was taught something else during the 70 years of the Soviet Union that the state is responsible for everything. Now they say you have to take responsibility for your health. (Sasha, 76-year-old man)

Generally, respondents believe that pandemic management measures have been less coordinated in Armenia compared to the Czech Republic, as during the initial outbreak period the Armenian government did not have a clear policy to fight against COVID-19 and demonstrated uncertainty. The differences between national responses were more pronounced at the level of implementation on the ground as opposed to the policy level. Karine, a 60-year-old woman whose son lives in the Czech Republic, revealed:

Starting from the very beginning I think it was stricter and better managed in Czechia. Of course, here we had the same measures later, but here people just don’t like following the rules. When you compare two countries, some differences become even more obvious. I know that in Prague you can’t go around without a mask, but here it seems that people stopped caring about safety. We need a stronger government response to control the situation. (Karine, 60-year-old woman)

Similarly, Armine, a 48-year-old woman whose brother has lived in the Czech Republic for about 10 years, revealed:

My older brother lives in the Czech Republic. According to him, after learning about the coronavirus, they were not allowed to gather with a friend in a cafe or stand on the street to talk. He was very surprised when I showed him the streets of our village and Yerevan, where very few people wore masks. We can enjoy our time especially in Yerevan like this, but I think people’s lives are the most precious things, so they are right to follow those rules. (Armine, 48-year-old woman)

Transnational families described their circumstances as unique compared to families that lived together, as the transnational families were aware, worried, and considerate of not only local regulations, but regulations in countries where other member(s) of their family reside as well.

### “Pandemic transnationalism”

The study of coronavirus practices shows that transnational families largely develop a special form of transnational social remittance, or as we term, “pandemic transnationalism.” The latter represents the transfer of ideas and exchange of caring practices across national borders related to coping with the pandemic. Mary’s husband is a jeweler in the Czech Republic. Reflecting on the initial months of the pandemic, Mary shared:

My husband has been constantly advising us what to do: put on a mask, keep your distance... do not go anywhere and avoid outside trips unless necessary. He is especially worried about me visiting the beauty salons. I did not go to the hairdresser for a year, he said the most dangerous places are beauty salons. I went secretly for the first time, otherwise he would be worried … For the last year our conversations are always about the same things: wear masks, do not go anywhere. (Mary, 48-year-old woman)

Non-migrants’ narratives reflect the multiple challenges they face when their family members are abroad during crises. Not only does having family abroad change family dynamics, but it also imposes alternative caring arrangements that migrants and non-migrants want to ensure for their family back home or in the host country. Karine, whose son lives in the Czech Republic, revealed:

If my son had not been abroad, I wouldn’t be so worried about coronavirus. But he was reminding me all the time about the rules, that’s why we were very alert. Also, we were worried about not getting sick, as it would have been hard for him to come to Armenia in these conditions, it is difficult to travel during the coronavirus. Not only him, but I was also checking the news all the time on the Czech Republic, the number of cases, new regulations. (Karine, 60-year-old woman)

“Pandemic transnationalism” is maintained through phone calls, emails, social media, and through sending necessary personal protective equipment, such as masks, gloves, and sanitizers, to protect families. During a pandemic, these “emergency” remittances are migrants’ ways of demonstrating their responsibility to other members of their families. No matter how little the difference is between gloves or masks sent from abroad or ones made and purchased in Armenia, the interviewees often underlined and spoke with enthusiasm of the “symbols of care” sent from abroad. Vahram, whose son lives in the Czech Republic, shared:

My son sent a box with N95 masks, because they told him that N95 or N99 is better than a usual mask. He keeps sending masks, gloves, hand sanitizers, and many vitamins. You can find vitamins also here, but I guess in this way he expressed his anxiety. (Vahram, 64-year-old man)

The dissemination and transfer of personal experience has proven to be a critical skill in dealing with the pandemic among transnational families. The spread of information through informal networks happens on a large scale, particularly in rural areas. International migration and exchange of practices also change the perception of what is wrong and what is right. The practices of migrants and their families are not always in line with the laws and regulations of either the host or sending countries. In her book on “Transnational Villagers,” Levitt (2001) presents the study of Dominican migrants in the US that adopt practices that are against US law, such as selling drugs over the counter that require a prescription in the United States. They see these minor infractions as rule bending, not rule breaking, and as necessary for their daily survival (Levitt, 2001, p. 120). Levitt (2001) finds that most migrants balance these against-the-law stances without dissonance. The perception of what is wrong and what is right is quite applicable to the practices of transnational families coping with the pandemic as well.

When in our village the virus started spreading, the first 2-3 infected people went to see the doctor, and the rest just used the same medicine to cure the virus, they were just taking the same medicines from the same pharmacy. When another person came to ask the pharmacist to give him the same medicine, the pharmacist asked him where he is from. The pharmacist later reported these details to the responsible bodies, and when they came and tested people, they saw that the majority of the population have the virus, and they immediately imposed quarantine. (Lilit, 33-year-old woman)

The research shows that practices are not only transferred from transnational family units to Armenia, but are also largely consumed in informal settings. A possible reason for the spread of informal practices was the lack of information provided by the state and distrust towards health regulations in Armenia. During the crisis, new information provided by migrant family members was quickly accepted by families in Armenia and spread among their social networks. Razmik, a 45-year-old man who survived COVID-19, relied on the advice of a relative based in Russia. Even though these practices came from an unverified source, he said he overcame the virus and shared the “prescription” with his friends and families:

My brother had coronavirus too, and he suggested that in Russia they have already developed the strategy, and he immediately contacted his doctor and asked him one more time for the exact procedure. Then I did the exact steps here … I was drinking a lot of liquid with lemon and, mostly, green tea. Almost 5 liters per day, and the vitamin complex in the morning. And on the second and third day I started taking amexine, and then on the fifth, sixth, and seventh day plaquenil... And for the eighth and ninth day I used antibiotics. I would advise people not to forget about drinking lots of water. (Razmik, 45-year-old man)

The research reveals that in case of pandemic transnationalism, both low- and highly- skilled migrants try to deliver the pandemic-coping practices of their country of residence to their families in Armenia. The status of a migrant in their country of residence can affect the dissemination of ideas through an informal setting. These differences between low- and high- skilled migrants are demonstrated in the cases of Armine, whose brother is a designer residing in Prague, and Sasha, whose son is a seasonal construction worker in Russia. Armine shared:

High school students in our village organized a big event dedicated to Women's Day, March 8^th^. My daughter had to perform too. The hall was full of parents and students. Days later I met the mother of one of my daughter's classmates in the store, she said she felt very weak and had a fever, the same was with me but I thought I had a little cold. My brother called from Prague, and said that it is very possible that I got infected with coronavirus. I heard about the coronavirus on TV, but I did not take it seriously, like everyone else. My brother urged me to go and take a test. I went to the hospital, but they said they did not have a test sample yet. He called and told me that he had consulted with a doctor he knew, and they advised me to drink water, take vitamins, eat healthy and more. I called also the mother of my daughter’s classmate and told her what to do. (Armine, 48-year-old woman)

To the question of whether their acquaintances immediately trusted the information Armine provided them, Armine replied “they know my brother very well, they know that he is an educated person... also the Czech Republic is more advanced, so they trusted and then they gave the advice to others.” In contrast to this story, Sasha shared:

My son does not have a permanent job, he is doing construction or repair work … Once, during a conversation, he learned that his uncle was sick with coronavirus. He said that every day they are advised on TV what to do to overcome the coronavirus. Then I called my brother and told him what he shared. My brother said he would do it, but I felt that he was kind of skeptical, maybe he thought that my son might have confused something, he would not remember well. (Sasha, 76-year-old man)

Thus, the interviews show that the status of the person who transmits information plays an important role in the spread of social remittances in an informal setting, as people believe that coronavirus-coping practices transmitted by highly-skilled migrants may be more accurate and less likely to be disinformation.

Research shows that “pandemic transnationalism” is a two-way process, as not only do migrants send remittances to their families, but rely largely on remittances sent from home as well. Cross-border flows of comments, narratives, and gossip create a collective sense of coronavirus-coping strategies. Migrants often try to overcome the pitfalls of their country of residence’s health policies. This was particularly relevant for families with members in Belarus, as they were anxious about their relatives and expected that the pandemic in Belarus would worsen in the near future. The level of anxiety about the coronavirus was higher among families who had elderly relatives living in Belarus, and they were more in favor of introducing additional health measures in Belarus. Laura and Anna, two women who both had parents living in Belarus, were extremely worried about the country’s approach towards coronavirus. They both said that they advised their parents to limit going out in public unnecessarily, visiting shops, and using public transport. Laura and Anna also shared that they sent medicines and antibiotics to their parents in case they needed them urgently. They sent the medicines with their friends and relatives to Belarus, as travel between both countries was not prohibited, and it was easier to find these medications in Armenia.

The reverse flows of pandemic transnationalism were also noticeable among some families who had members in the Czech Republic. Karine, an interviewee, shared that she asked her son to wash everything he buys from supermarkets with white vinegar as it acts as a disinfectant that can destroy the COVID-19 virus. As a result, in addition to all of the measures advised by the World Health Organization and the Czech government, he also followed advice provided by his mother.

The study also shows that the transfer of social remittances in times of pandemic is very much context-specific. The narratives that followed the COVID-19 outbreak and the nature of transnational connections dramatically changed once the war in Nagorno-Karabakh broke out in September 2020. As one of the respondents revealed:

When I think about it the dynamics have changed a lot. During the war it seemed that COVID-19 is a minor issue. Also, my daughter was mainly speaking about the situation on the border line, as it was a more urgent topic. Our 18- to 20-year-old youth was fighting on the frontline. During that period, we were dreaming about the times when corona was our only problem. (Araks, 64-years-old woman)

The interviews indicate that family-level social remittances or what we call “pandemic transnationalism” were replaced by collective remittances sent from abroad in support of the homeland.

### Mask, distance, shame, gatherings: “new normality” versus socio-cultural norms?

The general guidelines published by the World Health Organization are simple at first glance, including stipulations such as physical distancing, wearing a mask, keeping rooms well-ventilated, avoiding crowds, cleaning hands, and coughing into a bent elbow or tissue (World Health Organization, 2020a, b). However, these simple guidelines require individuals to make sense of a new situation and to negotiate it within the cultural and social norms of their society. Transnational families were caught in the middle of experiences, knowledge, and the state policies of at least two countries. The cases in this study often revealed that health restrictions were in conflict with certain cultural norms, which made it difficult for people to renounce these customs. The distribution of gender roles between men and women and the fulfillment of certain responsibilities towards relatives and friends were in direct conflict with the “new normality” imposed by the pandemic. In this regard, we looked at some of the rituals that migrant families struggled to renounce during the pandemic.

In the context of COVID-19, elderly people were considered the most at-risk group by the World Health Organization, but some elderly individuals maintained a conservative attitude towards safety guidelines despite the risks to their health. In that regard, Sasha, whose son lives in Russia, shared his story.

My neighbor living in front of me died last month. We were very close with our families. We could even share our last meal during our hard times. My son lives in Moscow, he said that despite the virus it wasn’t appropriate to not to go to his funeral, what would the neighbors think of us? Many people from the village participated. (Sasha, 76-year-old man)

The interviewees highlighted that it is not easy to continually negotiate coping strategies within the “new normality.” Expressing condolences to the relatives of the deceased during a funeral ceremony is accompanied by a handshake, which many people are ashamed to perform with gloves, or to regularly disinfect their hands in front of everyone. Armen, a 30-year-old man from Yerevan, revealed:

My brother lives in Prague. When he found out that one of our relatives had died in Yerevan, he ordered a wreath for him, and he told me to greet people with gloves. I went, but almost no one was there with gloves on. I was ashamed to wear them, I had not seen a couple of people for a long time. Some days later, I had a fever and thought I had a cold, but it turned out that I was infected with the coronavirus. (Armen, 30-year-old man)

Among interviewees, those whose family members migrated to Russia between the end of the 1990s and the beginning of the 2000s had developed dense ethnic networks, which made following pandemic guidelines more difficult. Araks, a 64-year-old woman whose daughter lives in Russia, shared the story of how her daughter was infected with coronavirus. She blamed the relatives of her daughter who invited her to a party, as the daughter felt uncomfortable refusing them:

I spoke with her the day before, and asked if she is comfortable about attending a party while the cases of coronavirus were rising in Moscow. She said that they are their relatives and she just can’t refuse. (Araks, 64-years-old woman)

Similarly, while comparing the behavior of people in Armenia and the Czech Republic, Lilit, a woman whose husband lives in the Czech Republic, found it is an “Armenian thing” to have a hard time following guideline, unless they are mandated by law:

As I understand it is not something strange for them in the Czech Republic to stay at home, avoiding any kind of guests. My daughter tells me that even in the same building mother and daughter do not visit each other. Even if two friends walk outside, they need to follow the rules of keeping the distance and wearing masks, because if you don’t keep [the distance] you will be fined around $500. The rules are there and you need to obey whether you want to or not. (Lilit, 33-year-old woman)

Another problem that arises in the cultural context is that family members and relatives are ashamed to speak out about being infected and keep it a secret as much as possible, as people change their attitude towards families with infected members by restricting contact and labeling. Silva, a 68-year-old woman, revealed:

My family and I have been living in Russia for 20 years. In January 2020 I came to Armenia to see my relatives, but I could not return because there were no flights and the borders were closed. My daughter works as a saleswoman in a shop there and she was infected [in Russia]. My husband said to not tell anyone about it and not to tell my relatives here that my daughter was infected, as they may change their attitude or feel differently towards us. (Silva, 68-year-old woman)

The study highlighted that there was a hidden masculine resistance to wearing masks, as in certain environments wearing marks was viewed as something shameful and unsuitable for a man. Masculinity and COVID-19-related health behaviors have been underlined by Capraro and Barcelo (2020), as they found men that live with more traditional masculine norms are more likely not to wear masks, as doing so is viewed as a sign of weakness. Due to these circumstances non-migrants find it difficult to follow the guidelines imposed by the state, but social remittances in the form of ideas that travel from countries with more restrictive measures have the potential of changing local dynamics.

My son has been studying marketing at one of the universities in Prague for three years... He told me that when the coronavirus spread there, everyone started wearing a mask. From the beginning, I was kind of drawn to it, but in the place where I live, it is not very common to crawl with a mask for a man or to catch a raincoat, they are perceived as a loss of manly qualities. But I got used to it a little bit, wearing a mask became normal for me. Every time he talked to me, he was insisting, explaining the benefits of avoiding the virus. But I have several conditions, so he is right, I gave up and now I wear the mask. (Alen, 58-year-old man)

The interviews show that one of the factors that make following COVID-19 guidelines difficult for transnational families is cultural and social norms that travel across borders. Social remittances can make a profound difference in adapting to a “new normality,” be it in favor of following safety guidelines or against.

## Conclusion

Based on Levitt’s (1998) original idea of social remittances, our qualitative study contributes to the migration literature by focusing on social remittances performed by transnational families in a time of pandemic. As we highlighted through the literature analysis, despite increased attention on the role of social remittances in a transnational context, social remittances in times of crises have remained an underdeveloped concept in migration literature. Our paper suggests that the COVID-19 (coronavirus) pandemic is an important lens for reexamining remittances, as it reveals several features and “new” meanings of cross-border transactions, such as forms, characteristics, and directions of social remittances, the role of social remittances in shaping a “new normality,” as well as the context-sensitivity of social remittances in times of global crises. Our analysis of Armenian transnational families with members in the Czech Republic, Russia, or Belarus leads to several significant observations.

First, we elaborated on a special type of social remittance that occurs in times of global crises that migrants and left-behind family members develop in an effort to care for each other and negotiate the “new normality.” We introduced the term “pandemic transnationalism” and defined it as the exchange of ideas and practices for coping with the COVID-19 (coronavirus) pandemic, which can affect the everyday behaviors of migrants and their families. The research shows that “pandemic transnationalism” interplays between the pandemic regulations of home and host countries, cultural and social norms of migrants’ home and host countries, and individual family circumstances. Analyses of transnational families that have different (Czech Republic) and rather similar (Russia, and to some extent Belarus) pandemic-coping policies to Armenia shows that the exchange of rules, practices, and ideas across national borders is especially important in countries where there is a low expectation and trust towards the local authorities due to a lack of information and weak measures to contain the virus. In these conditions, the role of interpersonal trust grows as part of everyday “tactics” in safety planning in order to effectively mitigate the effects of the pandemic and because of a perceived lack of institutional support. “Pandemic transnationalism” is also maintained through “emergency” remittances, which includes sending personal protective equipment, such as gloves, masks, medicine from abroad, as well as advice and “prescriptions” as symbols of care.

Second, the study shows that the circulation of safety rules and “good practices” during a pandemic actively shapes the everyday behaviors of migrants and their families, their level of perceived danger towards coronavirus, and their practical knowledge of safety measures. The study confirmed that through social remittances, transnational families negotiate roles, obligations, their everyday lives, and cultural practices, all of which play essential roles in the lives of transnational families (Barrett et al., 2014; Lacroix et al., 2016; Solari, 2019). Moreover, the exchange of ideas related to the pandemic are often utilized in collective settings with people beyond family members, such as friends, neighbors, and acquaintances. The research showed that collective consumption of social remittances is particularly applicable in rural communities. Migrants often act as a broker of ideas between their home country and the destination country by sharing “good practices” and advice with their families. Nevertheless, the collective consumption of social remittances also depends on the characteristics and status of the sender within the receiving society. Even though both high-skilled and low-skilled migrants transfer ideas that are developed within their host country, the practices transmitted by highly-skilled migrants have a greater degree of trust among recipients and a greater chance of being adopted by individuals in the home country.

Third, we have contributed to the further conceptualization of reverse remittances. The study shows that those left at home are not passive consumers of ideas, practices, and goods transferred from abroad. Moreover, social remittances during a pandemic are a two-way process, as not only migrants, but those left at home become senders of “emergency” remittances. Reverse flows of remittances during the pandemic differ from a “gift-giving” understanding of reverse remittances (Mauss, 1923–1924) that start as an obligation to reciprocate (Mazzucato, 2011) from the left-behind side (Boccagni, 2015) considering that those left behind often participate as an initiating side of the “emergency” remittances exchange process. The nuanced analysis of pandemic transnationalism highlights the shifting character of senders and recipients, as migrants are not always the ones conducting the sending of remittances, and families left behind are not always the recipients of social remittances. This feature is conditioned by the specificity of the pandemic, as both ends of a transnational family are equally affected by the crises.

Fourth, we have underlined the need for special attention to the receptivity of social remittances during crises and its relevance to cultural and social norms of the home country. The study highlighted several forms of social and cultural norms that actively influence the behaviors of transnational families during a pandemic and can function as potential barriers for negotiating a “new normality” within transnational families. The study demonstrated the tension between social remittances and COVID-19 safety measures, such as mask-wearing conflicting with gender stereotypes on masculinity, the need to avoid large events conflicting with the responsibility towards relatives, and the shame of being infected. In these conditions, many cultural norms brought from the country of origin continue to be practiced in pandemic conditions by migrants, which pushes families to have open and direct contact with others, ignoring even accepted safety rules and risks, especially in conditions where there is a presence of an ethnic network in the host country. Nevertheless, when COVID-19 safety measures are strict or well-established in the country of residence of one of the family members (such as the Czech Republic), there is a potential to change the dynamics of “new normality” rules for the rest of the family in the home country.

The final item that the research reveals is the context-sensitive and temporary nature of social remittances. The popularity of pandemic transnationalism changes in response to other crises that develop in home countries, such as the outbreak of war in Nagorno-Karabakh in September 2020. During these crises, remittance practices change their characteristics and take new forms, such as generating collective remittances in support of the homeland. Therefore, the ongoing COVID-19 pandemic makes it difficult to predict the course of future events. Further examination of social remittances during global crises has the potential to contribute to studies on transnational social protection practices (Lafleur & Romero, 2018; Schaab & Wagner, 2020). Moreover, the issue of informal practice exchange within transnational families and its effect on policy circulation and diffusion processes can also be of interest to researchers and policy makers in the context of limiting disinformation and promoting safety measures.

## Data Availability

The qualitative data used and analyzed during the current study is available from the corresponding author on reasonable request.
